# Chaperone-Mediated Autophagy in Neurodegenerative Diseases and Acute Neurological Insults in the Central Nervous System

**DOI:** 10.3390/cells11071205

**Published:** 2022-04-02

**Authors:** Haruo Kanno, Kyoichi Handa, Taishi Murakami, Toshimi Aizawa, Hiroshi Ozawa

**Affiliations:** 1 Department of Orthopaedic Surgery, Tohoku Medical and Pharmaceutical University, Sendai 983-8536, Japan; hozawa@tohoku-mpu.ac.jp; 2 Department of Orthopaedic Surgery, Tohoku University School of Medicine, Sendai 980-8574, Japan; khanda@med.tohoku.ac.jp (K.H.); taishi.murakami.q5@dc.tohoku.ac.jp (T.M.); toshi-7@ra2.so-net.ne.jp (T.A.)

**Keywords:** chaperone-mediated autophagy, autophagy, LAMP2A, Hsc70, neurodegenerative disease, Parkinson’s disease, Alzheimer’s disease, traumatic brain injury, spinal cord injury, central nervous system

## Abstract

Autophagy is an important function that mediates the degradation of intracellular proteins and organelles. Chaperone-mediated autophagy (CMA) degrades selected proteins and has a crucial role in cellular proteostasis under various physiological and pathological conditions. CMA dysfunction leads to the accumulation of toxic protein aggregates in the central nervous system (CNS) and is involved in the pathogenic process of neurodegenerative diseases, including Parkinson’s disease and Alzheimer’s disease. Previous studies have suggested that the activation of CMA to degrade aberrant proteins can provide a neuroprotective effect in the CNS. Recent studies have shown that CMA activity is upregulated in damaged neural tissue following acute neurological insults, such as cerebral infarction, traumatic brain injury, and spinal cord injury. It has been also suggested that various protein degradation mechanisms are important for removing toxic aberrant proteins associated with secondary damage after acute neurological insults in the CNS. Therefore, enhancing the CMA pathway may induce neuroprotective effects not only in neurogenerative diseases but also in acute neurological insults. We herein review current knowledge concerning the biological mechanisms involved in CMA and highlight the role of CMA in neurodegenerative diseases and acute neurological insults. We also discuss the possibility of developing CMA-targeted therapeutic strategies for effective treatments.

## 1. Introduction

Various important cellular functions, including maintaining viability, depend on protein homeostasis, namely, proteostasis [1]. Cellular proteostasis requires a constant balance between protein synthesis and degradation. In particular, maintaining cellular protein homeostasis is essential for long-lived post-mitotic cells, such as neurons [1,2,3]. Proteostasis is strongly associated with the recognition and removal of unwanted proteins to ensure protein quality control. Unwanted, damaged, misfolded, and aggregated proteins are mainly degraded by the ubiquitin–proteasome system (UPS) and the lysosome-dependent autophagic process [4].

Autophagy is an important cellular function that mediates the degradation of intracellular proteins and organelles in lysosomes. Autophagy plays a crucial role in cellular protein homeostasis. There are three forms of autophagy: macroautophagy, microautophagy, and chaperone-mediated autophagy (CMA) [5]. In brief, macroautophagy is characterized by the formation of double-membrane vesicles (autophagosomes) that fuse with lysosomes and degrade their contents. Microautophagy is characterized by lysosomal (vacuolar) or endosomal membrane dynamics to directly enwrap cytoplasmic components. In contrast, CMA is different from macroautophagy and microautophagy because it does not require the formation of vacuoles and only degrades selected individual proteins. CMA is a process in which the molecular chaperone heat-shock cognate protein 70 (Hsc70) targets the substrate with a KFERQ motif to the lysosome membrane [6,7]. Therefore, CMA plays a particularly important role in cellular proteostasis under various physiological and pathological conditions [8]. Indeed, nearly 30% of cytosolic proteins may potentially be targeted by CMA [9].

Selective protein degradation via CMA mediates cellular homeostasis under various stress conditions, such as starvation, hypoxia, and exposure to toxins [10,11,12]. Under such stress conditions, CMA degrades substrates selectively, thereby contributing to the elimination of altered proteins and recycling of amino acids. The timely degradation of specific proteins by CMA can regulate multiple cellular functions, such as glucose and lipid metabolism, DNA repair, and cellular reprograming [6]. Many studies have revealed that CMA dysfunction is related to the pathologies of various human diseases, such as cardiac diseases, liver diseases, cancer, and neurodegenerative diseases [4,6,13,14,15].

Importantly, changes in CMA activity play an important role in different pathologies in various human diseases affecting the central nervous system (CNS) [1]. In particular, CMA dysfunction leads to the accumulation of toxic protein aggregates in the CNS and is involved in the pathogenic process of various neurodegenerative diseases, such as Parkinson’s disease (PD), Alzheimer’s disease (AD), Huntington’s disease (HD), frontotemporal lobar degeneration (FTLD), and amyotrophic lateral sclerosis (ALS) [4,14]. In these diseases, many different pathogenic proteins have been identified as the substrates of CMA. Conversely, it has been also reported that CMA activity is upregulated in damaged neural tissue after traumatic brain injury (TBI) [16] and cerebral ischemia [17], suggesting the cytoprotective role of CMA against acute brain damage. In addition, a previous study showed that CMA activity is promoted in various neural cells following spinal cord injury (SCI) [18]. Therefore, CMA may play an important role in neuroprotective mechanisms following acute CNS injury.

In this review, we summarize current knowledge concerning the biological mechanisms involved in CMA and highlight the role of CMA in neurodegenerative diseases and acute neurological insults in the CNS. We also discuss the possibility of developing CMA-targeted therapeutic strategies to treat neurodegenerative disorders and acute CNS injury.

## 2. General Characteristics of CMA

Autophagy can be categorized into three main forms: macroautophagy, microautophagy, and CMA [5]. In macroautophagy, a newly-formed isolation membrane sequesters cytosolic proteins and organelles. This membrane then matures and seals to become a double-membrane vesicle called an autophagosome [5]. The contents of autophagosomes can be degraded by lysosome enzymes. During microautophagy, the intracellular components are directly captured by the lysosomal membrane via invagination [19,20]. The engulfed cargoes are then delivered into the lumen by vesicle scission for subsequent degradation [6,20,21]. Microautophagy generally participates in the constant removal of organelles and intracellular proteins [20]. In contrast, CMA does not require vesicle formation and involves cargo recognition and delivery of substrates to lysosomes instead [6]. CMA, in a unique way, selectively targets protein substrates and directly transports them into the lysosome lumen for degradation [8].

The function of CMA is presumed to be restricted to mammals and birds [22], although other autophagic pathways are conserved from yeast to mammals. Importantly, CMA activity has been detected in many different mammalian cell types, including fibroblasts [23], hepatocytes [24], astrocytes [18], primary neurons [25], macrophages [26], dendritic cells [26], T-cells [27], retinal cells [28], and a large array of cancer cells of different origins [13]. Furthermore, CMA studies have been performed with lysosomes isolated from the liver [29], spleen [30], different brain regions [25], and kidneys [31].

The levels of CMA activity vary depending on cell type and cellular conditions. CMA is maximally activated in most cells under stress conditions and contributes to selective degradation of unwanted or damaged proteins and organelles [8,9]. Selective degradation by CMA provides an important quality control mechanism to maintain intracellular proteostasis and avoid proteotoxicity [32,33]. During prolonged starvation, amino acids can be recycled and provide energy for cells [10,33]. During starvation-induced autophagy, macroautophagy can be activated by starvation for 30 min and reaches a peak after 4–6 h of treatment, while CMA is activated after 8–10 h and generally reaches a peak after 3 days of starvation [10,23,33].

The selectivity of CMA can be beneficial under conditions in which discrimination between different types of proteins for degradation is required. Activation of protein degradation via CMA during prolonged starvation will provide cells with free amino acids required to sustain protein synthesis [10,34]. In addition, activation of CMA during mild oxidative stress or after exposure to compounds that decrease proteostasis allows the selective removal of the proteins damaged or altered under these conditions [12]. Furthermore, selective protein removal through CMA has been shown to exert important regulatory functions in metabolic pathways and DNA repair pathways [24,35]. CMA is important for helping the immune system regulate the CD4+ T-cell response, as CMA selectively degrades negative regulators of T-cell activation [27]. Selective degradation via CMA is involved in the cell cycle and transcription by reducing the numbers of enzymes or transcriptional factors in cells [30,36,37].

## 3. Basic Molecular Mechanism of CMA

CMA is a selective degradation form of cytosolic proteins wherein the targeted protein substrates are directly translocated into the lysosomal membrane. To be CMA substrates, proteins must contain a specific targeting motif in their amino acid sequence.

**Figure 1:**
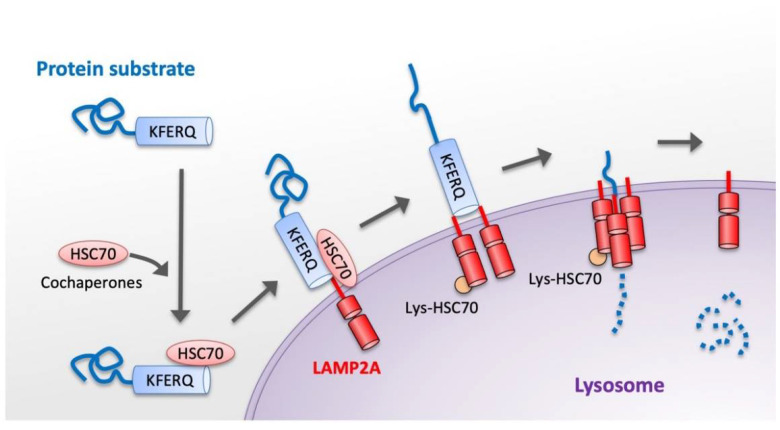
Process of protein degradation via chaperone-mediated autophagy. The KFERQ-like motif of the protein substrate is recognized by Hsc70 and its cochaperones. This complex binds lysosome-associated membrane protein type 2A (LAMP-2A). LAMP2A monomers are assembled into multimeric structures, forming the translocation complex. The substrate proteins are then unfolded and translocated through the lysosomal membrane, assisted by lysosomal Hsc70 (Lys-Hsc70). Finally, the protein is rapidly degraded within the lysosome and then the translocation complex is disassembled.

The basic process of CMA can be divided into the following steps: (I) substrate recognition and delivery to lysosome; (II) substrate binding to the lysosomal membrane; (III) substrate translocation through the lysosomal membrane; and (IV) substrate degradation in the lysosomal lumen (Figure 1) [4,6]. In the first step of the CMA process, cytosolic substrate proteins that contain the pentapeptide structure motif KFERQ are recognized by Hsc70, a cytosolic member of the Hsp70 chaperone family [7]. The protein substrate–chaperone complex is then delivered to the lysosomal surface. Second, the substrate complex binds to the lysosomal membrane, assisted by lysosome-associated membrane protein 2A (LAMP2A) [38]. LAMP2A monomers are then assembled into multimeric structures, forming the translocation complex that enables translocation of the substrates into the lysosomal lumen. Substrates can bind to LAMP2A in a folded state but they must be unfolded to be translocated to the lumen of the lysosome [39]. Third, the substrate proteins are unfolded and then translocated across the lysosomal membrane. There is a form of Hsc70 located within the lysosome (lys-Hsc70) that reinforces the translocation of the substrate. Finally, the substrate proteins are degraded rapidly by proteases inside the lysosome.

The activity of CMA is tightly regulated to maintain cellular proteostasis. The regulation of CMA depends on multiple aspects, such as the level of LAMP2A in the lysosome, the level of Hsc70, and the condition of the KFERQ-like motif of the substrate [1]. CMA activity also can be affected by the rate of assembly/disassembly of the translocation complex and the presence of lys-Hsc70 within the lysosomal lumen [4,8].

Compensatory mechanisms between CMA and other intracellular protein degradation systems are important for ensuring appropriate cellular proteostasis [40]. Although there are definite differences in the underlying molecular mechanisms between these two autophagic pathways, macroautophagy and CMA are closely connected during the lysosomal degradation process [41]. Indeed, macroautophagy can be upregulated under CMA-defective conditions [23]. In addition, the inhibition of macroautophagy can lead to activation of the CMA process [42]. It is noteworthy that UPS and the autophagy–lysosomal system are functionally coupled in the degradation of excess or damaged proteins to maintain cellular homeostasis and ensure neuronal survival [43,44]. Importantly, CMA and UPS collaborate to degrade the gene product of the regulator of calcineurin 1, whose overexpression has been linked to Down’s syndrome and AD neuropathology [45].

## 4. Experimental Research Tools to Assay CMA Activity

In this section, we describe the experimental research tools available for assaying CMA activity in vitro and in vivo. The methods commonly used to obtain information correlated with CMA activity as well as CMA functional assays are described.

### 4.1. Useful Analyses for Monitoring CMA Activity

Evaluating changes in the amounts of the main molecular components of CMA can be used as an indirect way of assessing CMA activity [46]. In addition, the number and distribution of CMA-active lysosomes can be analyzed to infer changes in CMA activity [46]. However, these analyses only provide the data correlated with CMA status. Thus, these analyses should be complemented with functional assays [47].

Immunoblotting and imaging for evaluating changes in the main CMA components are the most commonly used methods for assessing CMA activity. Since the lysosomal levels of LAMP2A are limited for CMA [48], changes in the abundance of the LAMP2A protein in lysosomes usually correlate with the activity of CMA. Therefore, in imaging evaluations, the presence of LAMP2A at the lysosomal membrane should be analyzed [47]. Immunoblotting for LAMP2A using lysosome-enriched fractions or at least a membranous cell fraction is more informative than that using total whole cell lysates [46].

Levels of lysosomal-hsc70 are also related to CMA activity [49]. However, hsc70 is one of the most abundant cellular chaperones, and the fraction located in lysosomes is a small amount. Therefore, immunoblotting for hsc70 in total cellular lysates is not informative for CMA [46]. Colocalization of hsc70 with lysosomal markers (e.g., LAMP1) can be used to detect CMA-active lysosomes [49]. The number of these lysosomes colocalized with hsc70 in proportion to the whole lysosomal pool increases when CMA is activated [12]. In addition, an electron microscopic analysis using immunogold staining for hsc70 can also provide information about the pool of CMA-active lysosomes [49].

In analyses using isolated lysosomes from cells or tissues, increased levels of well-known CMA substrates (e.g., GAPDH) can indicate a decreased activity of CMA [46]. In general, CMA substrates are rapidly degraded after translocation [6]. Thus, a comparison of the lysosomal levels of CMA substrates in cells or animals between models treated or untreated with inhibitors of lysosomal proteases (i.e., leupeptin) allows for the measurement of the flux in CMA values [24].

### 4.2. Functional Assays

Several functional assays enable the tracking of CMA activity over time in cells, tissues, and isolated organelles.

#### 4.2.1. Intracellular Protein Degradation Assessment

Approximately 30% of total cytosolic proteins can be degraded by CMA [9]. However, the actual fraction of the cytosolic protein degraded by CMA varies depending on the cell type and cellular conditions [6]. Therefore, measurement of the pool of cellular proteins that undergo degradation through CMA is a common method for determining the overall activity of the CMA pathway [46]. Pulse and chase experiments using a radiolabeled amino acid and inhibitors of either lysosomal proteases or other autophagic pathways can be used to discriminate proteins undergoing CMA degradation from those managed by other pathways [50].

#### 4.2.2. Photoconvertible CMA Reporters

In addition, a method of monitoring the lysosomal association of artificial fluorescent CMA reporters would also be useful for tracking substrate delivery and degradation through CMA [51]. Using photoconvertible fluorescent reporters [51], it is possible to track the association of the photoconverted protein with lysosomes in a different fluorescence channel. An increase in the number of fluorescent puncta per cell would be a good indicator of CMA activation [46].

#### 4.2.3. In Vitro Analyses of CMA Using Isolated Lysosomes

The cross-talk between different autophagic pathways makes it difficult to accurately assess CMA activity in intact cells [41]. Therefore, we must separately analyze all functional steps involved in the dynamic degradation process of CMA pathways [46,49]. The most reliable approach for analyzing CMA activity is obtained by in vitro reconstitution of CMA with isolated lysosomes [52].

Isolation of the specific fraction of lysosomes active in CMA allows for an analysis of the content of endogenous CMA substrates in the CMA compartments [47]. Isolated lysosomes also permit reconstitution of CMA in vitro to follow the steps involved in the CMA process—substrate binding, lysosomal uptake and lysosomal degradation [50]. Treatment with lysosomal protease inhibitors followed by incubation with the CMA substrate will allow for the measurement of the substrate bound and translocated into lysosomes (binding and uptake) [39]. By discounting the amount of substrate bound to lysosomes in which proteolysis has not been prevented, it would then be possible to calculate the uptake [39,53].

The isolated lysosomal fractions also allow for the direct comparison of changes in the content, post-translational modification, and organization of CMA components at the lysosomal membrane. Reductions in lysosomal LAMP2A or lys-hsc70 levels in isolated lysosomes are indicative of decreased CMA activity [54,55], whereas increases in LAMP2A levels suggest upregulation of CMA activity [56]. In addition, the ratio of lysosomal LAMP-2A assembled into a multimeric complex at a given time can be determined using blue native electrophoresis of isolated lysosomes and immunoblot for LAMP-2A [57].

## 5. Neurodegenerative Diseases and CMA

Neurons are post-mitotic cells and require efficient protein degradation machinery to maintain cellular homeostasis under stress conditions [58,59]. Impairment of the protein degradation process in the CNS causes aggregation of aberrant or damaged proteins, which is a distinct feature of many neurodegenerative diseases. Substantial evidence has been gathered that dysfunction of CMA is associated with different pathologies in various neurodegenerative diseases affecting the CNS [1,14]. In these diseases, various pathogenic proteins have been identified as the substrates of CMA, such as α-synuclein in PD [60], Tau protein in AD [61], huntingtin (Htt) in HD [62,63], and TDP-43 in ALS and FTLD [64,65].

### 5.1. Parkinson’s Disease

PD is one of the most common neurodegenerative disorders. The main pathological features of PD are gradual loss of dopaminergic neurons within the substantia nigra and aggregation of the protein α-synuclein in Lewy bodies. Numerous studies have shown that impairment of CMA is related to the main pathogenesis of PD [4,66]. In patients with PD, the level of LAMP2A protein is decreased in the brain, indicating that CMA activity is attenuated [67,68]. Many previous studies have suggested that inhibition of the CMA degradation pathway causes the accumulation of α-synuclein, which is associated with the gradual loss of dopaminergic neurons [8]. Importantly, the mutant forms A53T and A30P of α-synuclein identified in familial PD cannot be degraded by CMA. Furthermore, these mutant forms tightly bind to LAMP2A at the lysosomal membrane and consequently inhibit the normal degradation of other CMA substrates in vitro [60,69].

G2019S mutation in leucine-rich repeat kinase 2 protein (LRRK2) can be a pathological cause of familial PD [70]. The G2019S mutant inhibits the dynamic assembly of the CMA translocation complex at the lysosomal membrane, causing the dysfunction of CMA in a mouse model of PD and in the brains of mutant LRRK2 PD patients [25]. In addition, the pathogenic mutant forms of LRRK2 binds to cytosolic Hsc70 and interacts abnormally with CMA components, blocking the degradation of other CMA substrates and neuronal protein homeostasis in vitro [25,71].

Ubiquitin *C*-terminal hydrolase L1 (UCH-L1) physically interacts with LAMP-2A, Hsc70 and Hsp90 and is involved in the regulatory mechanism of the CMA pathway [72]. In a previous study, the I93M mutant form of UCH-L1 was identified in a single PD family [73]. It has also been reported that the I93M mutation in UCH-L1 abnormally enhanced interaction with the cytosolic region of LAMP2A, inhibiting the CMA pathway in vitro [74]. Furthermore, the expression of the I93M mutant form of UCH-L1 in mammalian cells induced the CMA inhibition-associated increase in the amount of α-synuclein [74]. These findings suggest that aberrant interaction of the I93M mutant form of UCH-L1 with CMA machinery might underly the pathogenesis of PD associated with the aggregation of α-synuclein.

Parkinson’s disease protein 7 (PARK7), also known as DJ-1, is a multifunctional protein involved in a variety of cellular activities, including oxidation resistance [75]. PARK7/DJ-1 has an important role in maintaining mitochondrial homeostasis [75]. It has been reported that a mutation in the DJ-1 gene mediates autosomal recessive and early forms of PD [76]. DJ-1 deficiency accelerated the degradation of LAMP2A in lysosomes, leading to the aggregation of α-synuclein [77]. In contrast, DJ-1 was able to inhibit the accumulation of α-synuclein by regulating CMA [78].

Overall, various molecular mechanisms causing dysfunction of CMA are considered to underlie the pathogenesis of PD. However, the pathological mechanisms associated with CMA remain largely unclear. Further research will be needed to clarify the relationship between the CMA process and actual pathologies of PD.

### 5.2. Alzheimer’s Disease

AD is the most common neurodegenerative disease in the elderly. The main pathogenesis of AD is amyloid-β plaque formation and Tau aggregation caused by the impairment of protein homeostasis. Several proteins related to AD have been identified as CMA substrates. The CMA degradation of these protein substrates was shown to be impaired in patients with AD [45,61,79]. The progressive accumulation of amyloid-β oligomers is a central toxic event in AD [80,81]. A recent study showed that tagging amyloid-β oligomers with multiple KFERQ motifs promoted their entry into endosomes and lysosomes, protecting human primary cultured cortical neurons from neurotoxicity [82].

Amyloid precursor protein (APP) is an important pathogenic molecule in AD because it can be processed to produce amyloid-β [83]. APP contains a KFERQ-like motif at its C terminus. This motif is important for the normal processing and degradation of APP to prevent the accumulation of APP-*C*-terminal fragments [84]. A recent study revealed that APP is a CMA substrate that binds to Hsc70 [85]. The inhibition of CMA degradation of APP enhances its cytotoxicity. Furthermore, activation of CMA by Hsc70 overexpression or Metformin reduced the accumulated brain amyloid-β plaque levels and reversed the molecular and behavioral AD phenotypes in a mouse model of AD [85].

Tau is a cytosolic protein that normally stabilizes microtubules in neuronal cells. Tau protein has CMA-targeting motifs and can be degraded by the CMA pathway [79]. Aggregation of mutant Tau proteins resulting in Tau hyperphosphorylation and the formation of neurofibrillary tangles is a hallmark of AD and related tauopathies [86,87]. In addition, the mutant Tau proteins can interact abnormally with LAMP2A and inhibit translocation into the lysosome lumen, impairing CMA activity [79].

Regulator of calcineurin 1 (RCAN1) has been shown to be a substrate of CMA [45] and is elevated in patients with AD [88]. RCAN1 is an inhibitor of calcineurin-dependent dephosphorylation of Tau proteins. Importantly, CMA activity can be inhibited by increasing the level of RCAN1, thereby impairing the degradation of other substrates of CMA [45].

### 5.3. Huntington’s Disease

HD is a late-onset neurodegenerative disorder characterized by uncontrolled movement, dementia, and emotional disturbance. HD is a dominantly inherited disease caused by the accumulation and aggregation of mutant Htt protein in striatal and cortical neurons. Htt contains an abnormally expanded *N*-terminal polyglutamine (polyQ) tract [62,63,89]. Dysfunction of Htt degradation is suggested as the main pathogenesis of HD. Previous studies have shown that CMA is involved in the degradation of mutant Htt in cellular and mouse models of HD [62]. Htt harbors a putative KFERQ motif and interacts with the key components of CMA, Hsc70, and LAMP2A. In addition, mutant Htt with an expansion of the polyQ tract displays an impaired uptake by CMA in vitro [89].

Not only CMA but macroautophagy is involved in the degradation of Htt [90]. Htt can bind to both LAMP2A and the macroautophagy-related protein Atg7 in the degradation process [89,91,92]. CMA activity is reportedly upregulated in cellular and animal models of HD in the initial stage of the disease. However, the activity of CMA decreases during the late stage of the disease [91]. These findings suggest that the early increase in CMA activity may be a compensatory regulation in response to the inefficiency of macroautophagy. The decline in the level of lysosomal LAMP2A indicates that there is a loss of CMA function in the late phase of HD [91].

### 5.4. Amyotrophic Lateral Sclerosis and Frontotemporal Lobar Degeneration

ALS and FTLD are neurodegenerative diseases with many similar clinical and pathological features [93]. Transactivation response DNA-binding protein 43 kDa (TDP-43) is a ribonuclear protein regulating many aspects of RNA metabolism. The accumulation of TDP-43 *C*-terminal fragments in neuronal cells is frequently detected in patients with ALS and FTLD [65]. Thus, TDP-43 accumulation is widely considered a hallmark of these diseases. TDP-43 protein contains a KFERQ-like motif binding to Hsc70 and can be degraded by the CMA process [94,95]. Hsc70 expression was reportedly reduced in lymphomonocytes of sporadic ALS patients and contributed to TDP-43 accumulation [64]. A mutation in the KFERQ-like motif in TDP-43 can disrupt its degradation via CMA, inducing the accumulation of TDP-43 and the inhibition of CMA in cultured cells [94]. CMA can help control the turnover of the physiological and pathological forms of TDP-43 [94].

## 6. Acute Neurological Insults and CMA

### 6.1. Traumatic Brain Injury

TBI initiates a cascade of multiple pathophysiological processes, including the degradation pathway of aberrant proteins, such as macroautophagy and UPS [96,97]. These degradation systems are considered to be activated in response to various stress conditions after TBI. Decreasing toxic aberrant proteins via the autophagic process may provide a neuroprotective effect following TBI [98,99]. Importantly, a previous study showed that LAMP2A expression increased in neurons and proliferated microglia in a rat model of TBI [16]. In that study, the upregulation of LAMP2A occurred from 3 to 15 days following TBI. Another study using a mouse model of TBI also demonstrated that LAMP2A expression was upregulated in the injured brain [100]. These findings suggested that the CMA pathway can be activated in damaged neural tissue after TBI.

A recent study demonstrated that annexin A1 peptide Ac2-26 activated the CMA process to degrade IKKβ and consequently reduced TNF-α expression in microglial cultures [101]. These findings suggest that there is an anti-inflammatory mechanism associated with the CMA process in microglia [101]. Interestingly, silent information regulator 1 (Sirt1) activated CMA by upregulating DnaJ heat-shock protein family member B1 (Dnajb1) expression and consequently attenuated astrocyte activation and neuronal loss after TBI in mice [100]. Taken together, these findings suggest that the activation of the CMA pathway following TBI might exert a neuroprotective effect of attenuating inflammatory reactions and reducing neural tissue damage in the injured brain [100]. However, the actual function of CMA in TBI remains largely unknown. Further studies will thus be needed to elucidate the pathophysiological and neuroprotective mechanisms of CMA following TBI.

### 6.2. Cerebral Ischemia

Ischemic cerebral stroke is one of the leading causes of death and morbidity in humans. Previous studies have suggested that over-activation of autophagic pathways exerts a neuroprotective effect in ischemic brain injury [102,103]. Hsc70 and Hsp40 are reported to be synergistically expressed in the neurons of vulnerable areas in response to sub-lethal ischemia [104]. The combination of Hsc70 and Hsp40 suppresses aggregate formation and apoptosis in neurons [105]. Another study showed that the upregulation of LAMP-2A expression and the accumulation of LAMP-2A-positive lysosomes were induced under ischemic conditions in neuronal cells in vitro [17]. In an animal model of cerebral ischemia, LAMP-2A expression was slightly decreased until two days after ischemia and then the level increased significantly seven days after ischemia [17]. These findings suggest that CMA may be activated under ischemic conditions in the brain and may facilitate neuronal survival.

Blocking LAMP-2A expression with siRNA increased neuronal cell death after brain ischemia. [17]. In addition, the administration of mycophenolic acid, a potent CMA activator, rescued hypoxia-mediated cell death in a brain ischemia model. Furthermore, a membrane-permeable peptide that specifically binds to cyclin-dependent kinase 5 (CDK5) with a CMA targeting motif (Tat-CDK5-CTM) can promote the degradation of CDK5, reducing neuronal cell death [106]. In addition, Tat-CDK5-CTM also reduced the infarction area and neuronal loss and improved the neurological functions in a cerebral infarction mouse model [106]. Taken together, these findings suggest that promoting CMA activity may lead to the acceleration of the removal of damaged protein, thereby contributing to the survival of neurons after cerebral ischemia.

### 6.3. Spinal Cord Injury

Degradation of dysfunctional intracellular components via the autophagic process is a crucial step in maintaining cellular homeostasis in response to various forms of stress, including nutrient deprivation, hypoxia, reactive oxygen species, DNA damage, and endoplasmic reticulum (ER) stress [15,99,107,108]. Many previous studies have provided experimental evidence that autophagy is an essential cytoprotective pathway for reducing secondary neural tissue damage and functional impairment after SCI [99,109,110,111]. We previously reported that LAMP2A protein expression was significantly upregulated in damaged neural tissue after SCI in mice [18]. The expression of LAMP2A was increased in various neural cells, such as neurons, astrocytes, oligodendrocytes, and microglia, in the injured spinal cord [18]. These results indicated that CMA was activated in damaged neural tissue following SCI. Interestingly, our results also showed that the number of LAMP2A-expressing cells increased from 24 h and peaked at 3 days, lasting for at least 7 days after injury. The time course of LAMP2A expression is similar to that of apoptosis after SCI [112,113,114]. Apoptosis is considered a major cause of secondary damage following SCI [112,114]. Therefore, CMA activity might be regulated in response to secondary neural tissue damage.

A previous study showed that histone deacetylase-6 (HDAC6) has a molecular function of inducing Hsp90 deacetylation and increasing the interaction between LAMP2A and Hsp90, thereby upregulating CMA activity [115]. Another study showed that a deficiency in HDAC6 hindered CMA activity to resist oxidative stress in vitro [116]. In addition, inhibition of HDAC6 accelerated reactive oxygen species (ROS) generation and neuronal apoptosis in response to hypoxia–ischemia [116]. Importantly, both HDAC6 and LAMP2A expressions have been shown to be upregulated in a mouse model of SCI [116]. Taken together, HDAC6 may have an important role in the regulation of CMA activity and may be a potential therapeutic target for the effective treatment of SCI. Further studies will be needed to clarify the pathophysiological and cytoprotective mechanisms of CMA after SCI.

In summary, previous studies have shown evidence that CMA activity can be upregulated in damaged neural tissue following various types of acute neurological insults, such as cerebral infarction [17], TBI [16], and SCI [18]. Therefore, the CMA pathway may play an important biological role not only in neurogenerative diseases but also in acute neurological insults to the CNS.

## 7. Therapeutic Potential of CMA for Neurodegenerative Diseases

Major neurodegenerative diseases are generally caused by the accumulation of aberrant proteins, as described above. Aberrant proteins, such as α-synuclein and LRRK2 in PD, RCAN1 and Tau protein in AD, Htt in HD, and TDP-43 in ALS and FTLD, are the substrates of CMA [4,14]. Thus, the upregulation of CMA activity has therapeutic potential for treating neurodegenerative diseases caused by misfolded proteins [15]. As a therapeutic approach, CMA activity can be modulated by various molecular mechanisms, such as changing the LAMP2A level in lysosomes, changing the Hsc70 level, and changing the condition of the KFERQ-like motif.

Many studies have suggested that enhancing LAMP2A expression to upregulate the activity of CMA can be an important therapeutic target. A previous study demonstrated that recombinant adeno-associated virus augmenting the LAMP2A level protected dopaminergic neurons in the substantia nigra from α-synuclein-induced degeneration [117]. In addition, it has also been reported that various compounds, such as geldanamycin [118], 6-aminonicotinamide [119], glucose-6-phosphate dehydrogenase inhibitor [119], silymarin [120], chronic caffeine [121], manganese [122], trehalose [123], b-asarone [124], and other compounds extracted from natural medicinal plants [125], or even combination treatments with bortezomib and suberoylanilide hydroxamic acid (SAHA) [126], can increase LAMP2A levels and activate the CMA pathway. However, these compounds are not able to specifically regulate the CMA pathway and have many other targets. Thus, it is important to develop selective CMA modulators that can be used to manage human diseases.

Recent studies have revealed a novel molecular mechanism involving the effect of deacetylase and methyltransferase enzymes on the activity of chaperones in the CMA process. It was also reported that histone deacetylase 10 (HDAC10) deacetylates Hsc70 and upregulates the CMA pathway in vitro [127]. In addition, HDAC10 knock-out in cells results in the accumulation of LAMP2A-positive lysosomes around the nucleus, activating CMA to degrade a well-known CMA substrate, GAPDH [128]. These findings suggest therapeutic potential in the regulation of Hsc chaperones for CMA activation.

Another therapeutic approach involves the modification of the condition of the KFERQ-like motif of pathological proteins to make them suitable for degradation via the CMA pathway. A recent study showed that tagging amyloid-β oligomers with multiple KFERQ motifs promoted their entering endosomes and lysosomes, thereby protecting human primary cultured cortical neurons from neurotoxicity [82]. In addition, the use of an adaptor containing two copies of polyQ binding sequences and two different KFERQ motifs specifically directed mutant Htt to CMA degradation, ameliorating symptoms in a HD disease model [62]. An artificial peptide containing two CMA recognition motifs fused to two copies of the polyglutamine-binding peptide 1 (QPB1) sequence enables Htt to be degraded by CMA, ameliorating Htt aggregation and toxicity [62]. Interestingly, a novel antibody containing an KFERQ-like motif was able to recognize TDP-43 and targeted it to lysosomes for CMA degradation [129]. These findings suggest that modification of the condition of the KFERQ-like motif of aberrant proteins may be a new therapeutic strategy for treating neurodegenerative diseases.

The chemical enhancement of CMA can protect cells from oxidative stress and proteotoxicity. Signaling through retinoic acid receptor alpha (RARα) inhibits CMA activity. Synthetic derivatives of all-trans-retinoic acid can specifically neutralize this inhibitory effect [32]. Recently, it was also reported that humanin has molecular functions to antagonize endogenous CMA inhibitors and promote interaction between the CMA chaperone Hsp90 and the CMA receptor LAMP2A. Humanin and its analogs can enhance the CMA pathway by increasing substrate binding and translocation into lysosomes and exert cytoprotective effects against hypoxia-induced cell death [130]. Another study found that metformin, a drug commonly prescribed for type 2 diabetes, can activate the CMA pathway and prevent the accumulation of amyloid-β plaque in an animal model of AD [85].

Different protein degradation systems are wired to maintain cellular proteostasis under various physiological and pathological conditions. Protein degradation via CMA is achieved through the lysosome-based autophagy system and therefore interacts with macroautophagy and UPS [4,40,131]. Thus, a therapeutic approach that activates the CMA, macroautophagy, and UPS pathways should provide complementary or synergistic effects in restoring protein homeostasis [40]. However, the molecular mechanism involved in the interplay between these different protein degradation pathways has not been fully elucidated. Exploring the mechanisms underlying the cross-talk between CMA, macroautophagy, and UPS may facilitate the development of an effective therapeutic strategy to restore proteostasis in various neurodegenerative diseases.

## 8. Therapeutic Potential of CMA for Acute Neurological Insults

Following acute neurological insults to the CNS, including cerebral infarction, TBI and SCI, secondary injury can be induced by various molecular mechanisms, such as oxidative stress and neuroinflammation in the brain and spinal cord [132,133]. Such secondary injury is involved in multiple pathologies associated with neural cell death and neurodegeneration, aggravating the initial tissue damage of the CNS [112,113,132]. The secondary damage can be a potential therapeutic target for effective treatment of acute neurological insults to the CNS. Many previous studies have shown that activation of the autophagic process can exert a neuroprotective effect against secondary damage after acute CNS injury [99,134]. Notably, several studies have suggested that the upregulation of CMA activity may help reduce secondary neural tissue damage following acute neurological insults to the CNS [6]. As mentioned above, mycophenolic acid administered to activate the CMA pathway rescued hypoxia-mediated cell death after brain ischemia in an in vitro model [17]. In addition, Tat-CDK5-CTM increases the CMA degradation of CDK5, reducing the infarction area and neuronal loss and improving the neurological functions in a mouse model of cerebral infarction [106]. Furthermore, HDAC6 can regulate Hsp90 acetylation to enhance CMA activity and exert a neuroprotective effect after SCI in mice [116]. The upregulation of Dnajb1 expression induced by Sirt1 activated CMA and consequently reduced neuronal loss in a mouse model of TBI [100]. Therefore, enhancing the CMA pathway to remove toxic proteins may be a novel therapeutic approach to reduce secondary neural tissue damage after acute neurological insults.

Acute neurological insults in the CNS damage different types of neural cells, such as neurons, oligodendrocytes, astrocytes, and microglia. Such damage to these neural cells causes complex pathophysiological processes, including extensive neuronal cell loss, axonal injury, demyelination, and destruction of the blood–brain/spinal cord barrier [132,133]. Importantly, the activity of CMA is increased not only in neurons but also in microglia at the lesion site after TBI and SCI [16,18]. Microglia play various important roles in neuroprotection and neuroinflammation following acute CNS injury [135,136,137]. As described above, annexin A1 peptide enhances the CMA activity to degrade IKKβ and consequently reduces the TNF-α expression in microglia, suggesting an anti-inflammatory mechanism associated with CMA [101]. In addition, the activity of the CMA pathway is also upregulated in astrocytes and oligodendrocytes after SCI in mice [18]. CMA activation has been shown to reduce α-synuclein accumulation in astrocytes and oligodendrocytes in vitro [138,139]. The reduction in α-synuclein aggregation in the injured spinal cord has been reported to provide neuroprotective effects, attenuating axonal damage, neuronal loss, and neuroinflammation following SCI [140]. Previous studies have also suggested that autophagic activity contributes to the survival of oligodendrocytes and prevention of myelin loss after SCI [141]. Taken together, modulation of CMA activity in various type of glial cells may affect multiple pathophysiological processes following acute neurological insults in the CNS. It is important to determine the molecular mechanisms underlying the interaction between CMA and various different pathologies in the damaged CNS.

Many studies have suggested that depositions of aberrant proteins, such as amyloid-β and Tau protein, are observed in the brains of patients after TBI [142]. The pathological accumulation of aberrant proteins after TBI can be a major risk factor for several progressive neurodegenerative diseases, such as AD and PD [142,143]. The aggregation of amyloid-β is accelerated in injured brains, and amyloid-β plaques can be a pathological cause of neurodegenerative diseases in chronic-stage TBI [144,145]. TBI can also reportedly induce the aggregation of Tau proteins, which is a common feature of several neurodegenerative disorders [146]. Importantly, enhancement of the CMA pathway can decrease the accumulation of amyloid-β and Tau proteins in the brain [4,6,85,117,147]. Thus, the upregulation of CMA may aid in removing toxic aberrant proteins causing late-onset neurodegeneration after TBI.

## 9. Concluding Remarks and Future Perspectives

In the past decade, the regulatory mechanisms involved in the CMA degradation pathway have become clearer, expanding our understanding of the importance of CMA in cellular functions [4,6,8]. There is increasing evidence that CMA dysfunction is associated with different pathologies in neurodegenerative diseases in the CNS [1,4,6,14,15]. Important pathogenic proteins have been identified as the substrates of CMA, such as α-synuclein in PD [60], Tau protein in AD [61], huntingtin (Htt) in HD [62,63], and TDP-43 in ALS and FTLD [64,65]. However, most previous studies related to CMA in the CNS have focused on neurodegenerative diseases rather than acute neurological insults, such as TBI and SCI [6,14]. The CMA function in acute neurological insults in the CNS is still an immature research field and limited evidence has been published thus far. As mentioned above, CMA activity is likely to be upregulated in damaged neural tissues after acute CNS injury [16,18,100,116]. The actual function of CMA activation following acute injury of the brain and spinal cord remains unknown. Therefore, further studies will be necessary to assess the possible association of CMA with acute neurological insults in the CNS.

Various compounds have been reported to increase LAMP2A levels and activate the CMA pathway [118,119,120,121,122,123,124], as described above. However, these compounds cannot selectively regulate the CMA pathway. Therefore, it is important to develop selective CMA modulators that can be used for clinical treatment of human diseases [4]. The development of pharmacological selective CMA modulators will be a crucial step towards the implementation of therapeutic strategies aimed at improving cellular homeostasis through the regulation of CMA in the CNS.

Several currently available FDA-approved drugs and natural products have been found to promote CMA activity [85,121,148,149]. These drugs and products that enhance CMA might be able to be translated into novel clinical applications. Clinical trials involving autophagy as a therapeutic target for neurodegenerative diseases have focused on macroautophagy, not CMA [150,151]. No clinical trial has yet targeted CMA for the treatment of any neurodegenerative diseases. It will be important to develop new drugs that can selectively modulate CMA in target organs to maximize the therapeutic effect and minimize toxicity in clinical use.

Modulation of the CMA pathway may be promising for developing novel treatments of neurodegenerative diseases as well as acute neurological insults in the CNS. However, this area of research remains largely unexplored. Further efforts are needed to clarify the actual biological function of CMA in various pathophysiological processes in the brain and spinal cord. It is also important to elucidate the interaction between CMA and other protein degradation systems. Future research on these issues will aid in the development of novel clinical applications of CMA for the treatment of neurodegenerative diseases and acute neurological insults in the CNS.
